# Mapping binary copolymer property space with neural networks†
†Electronic supplementary information (ESI) available: Training data (before and after calibration), co-polymer optoelectronic property space data and associated SMILES for all copolymer compositions, machine learning model and training parameters, Python module (pychemlp, ref. 61) for recreating fingerprints, model and data. Raw data can be accessed freely *via* a GitHub repository (https://github.com/ZwijnenburgGroup/2019-polymer-neural-network). See DOI: 10.1039/c8sc05710a


**DOI:** 10.1039/c8sc05710a

**Published:** 2019-04-01

**Authors:** Liam Wilbraham, Reiner Sebastian Sprick, Kim E. Jelfs, Martijn A. Zwijnenburg

**Affiliations:** a Department of Chemistry , University College London , 20 Gordon Street , London , WC1H 0AJ , UK . Email: m.zwijnenburg@ucl.ac.uk; b Department of Chemistry and Materials Innovation Factory , University of Liverpool , Crown Street , Liverpool , L69 7ZD , UK; c Department of Chemistry , Molecular Sciences Research Hub , Imperial College London , White City Campus, Wood Lane , London , W12 0BZ , UK

## Abstract

The extremely large number of unique polymer compositions that can be achieved through copolymerisation makes it an attractive strategy for tuning their optoelectronic properties. However, this same attribute also makes it challenging to explore the resulting property space and understand the range of properties that can be realised. In an effort to enable the rapid exploration of this space in the case of binary copolymers, we train a neural network using a tiered data generation strategy to accurately predict the optical and electronic properties of 350 000 binary copolymers that are, in principle, synthesizable from their dihalogen monomers *via* Yamamoto, or Suzuki–Miyaura and Stille coupling after one-step functionalisation. By extracting general features of this property space that would otherwise be obscured in smaller datasets, we identify simple models that effectively relate the properties of these copolymers to the homopolymers of their constituent monomers, and challenge common ideas behind copolymer design. We find that binary copolymerisation does not appear to allow access to regions of the optoelectronic property space that are not already sampled by the homopolymers, although it conceptually allows for more fine-grained property control. Using the large volume of data available, we test the hypothesis that copolymerisation of ‘donor’ and ‘acceptor’ monomers can result in copolymers with a lower optical gap than their related homopolymers. Overall, despite the prevalence of this concept in the literature, we observe that this phenomenon is relatively rare, and propose conditions that greatly enhance the likelihood of its experimental realisation. Finally, through a ‘topographical’ analysis of the co-polymer property space, we show how this large volume of data can be used to identify dominant monomers in specific regions of property space that may be amenable to a variety of applications, such as organic photovoltaics, light emitting diodes, and thermoelectrics.

## Abstract 2

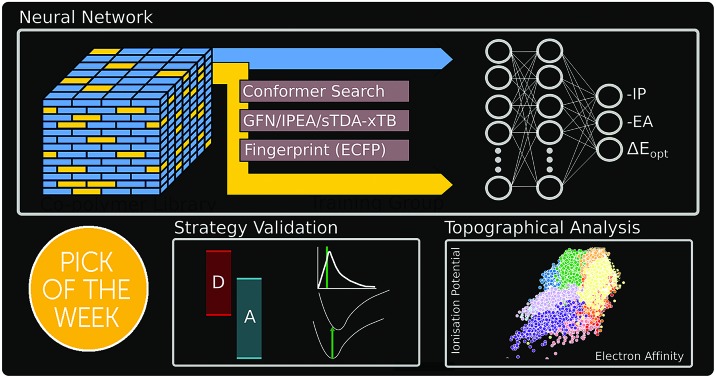
We map the property space of binary copolymers to understand how copolymerisation can be used to tune the optoelectronic properties of polymers.

## Introduction

Conjugated polymers are a highly versatile class of organic materials that can be used in a wide variety of applications such as photovoltaics,^1^–^5^ light-emitting diodes,^6^,^7^ field-effect transistors,^8^ batteries,^9^ supercapacitors,^10^ thermoelectrics,^11^,^12^ and photocatalysts.^13^–^18^ All of these applications exploit a combination of the optoelectronic and/or redox properties of the polymers, the earth-abundance of their constituents, and the relatively facile tunability of polymer properties. Generally, property tuning of conjugated polymers is performed through copolymerisation; combining different building blocks to yield a repeating motif, which is replicated to form the polymer chain. The properties of the resulting copolymers arise from a combination of those of the building blocks, although the exact connection between the two or between the properties of the copolymer and the related homopolymers is not clear. Models that aim to explain this connection for the optoelectronic properties in terms of the donor and acceptor character of building blocks have been proposed in the literature, but these are generally qualitative in nature.^19^–^21^

While an attractive attribute of polymer chemistry, the ability to both tune polymer properties through copolymerisation, and to explore their compositional space presents a dimensionality problem that arises from the large number of available monomers and is exaggerated with increasing copolymer complexity. To illustrate this numerically, consider a pool of 500 different monomers. Combining these monomers in all possible ways results in 125 250 binary copolymer compositions, increasing to over 250 000 when we consider that each repeat unit (if asymmetric) has two isomers. With more complex repeat units, *i.e.* three- and four-component copolymers,^4^,^5^ we arrive at billions of possible combinations. From a materials design standpoint, these astronomically large numbers make it impossible to explore the copolymer compositional space experimentally, even with high-throughput robotic synthesis and characterisation techniques, or computationally, particularly with more complex polymer repeat units, using standard approaches based around Density Functional Theory (DFT).

Naturally, we can overcome the copolymer dimensionality problem with a fast enough way of determining relevant properties for known copolymer compositions. A first step towards this was a move from DFT to semi-empirical methods, which allowed for the screening of short oligomers for high efficiency organic photovoltaic materials.^22^–^24^ In recent years, machine-learning techniques have emerged as a promising way of tackling analogous problems in other areas of organic and inorganic materials design,^25^–^33^ and conceptually could allow for the exploration of much larger compositional spaces, unlimited by polymer length. In this context, (supervised) machine learning involves ‘training’ a model with examples of molecules/materials for which the properties are known. Once trained, the model essentially acts as a function able to map molecular structure and/or composition to material properties. However, use of these techniques is often prohibited by the requirement for large amounts of clean, high quality, data with which to conduct training. We could obtain training data from electronic structure calculations, where, in the context of organic materials, DFT is the standard. However, DFT is simply too computationally intensive to use for large numbers of conjugated copolymers, where representative oligomer models can contain upwards of 150 atoms. Indeed, recent work^34^ on non-conjugated polymers using Gaussian Process Regressors trained using DFT data as input highlighted the challenge of exploring a wide chemical space with large numbers of possible compositions, as well as restrictions on the type of machine learning algorithms that are feasible, due to the limited size of the training data-set that is computationally affordable. Until recently, using semi-empirical methods, as discussed above, to generate this data could mean significantly reduced performance of a given machine learning model due to their lower accuracy with respect to DFT.^35^ However, we recently showed that optoelectronic properties calculated with xTB^36^–^38^ – a recently developed family of density functional tight binding methods – calibrated to a small, representative subset of (time-dependent-) DFT-derived results – provides highly accurate copolymer optoelectronic properties with computational cost reduced by at least three orders of magnitude relative to DFT.^35^ Further, we used the resulting high-throughput approach to demonstrate the weak dependence of the predicted properties on the exact polymer conformation.^39^ In turn, these two observations suggest that (i) xTB can be used to generate DFT-quality training data and (ii) 3D structural models of polymer chains may not be necessary for the prediction of optoelectronic properties (*i.e.* we can ignore conformation effects while focussing only on composition, see below), permitting the use of 2D molecular representations as descriptors.

**Fig. 1:**
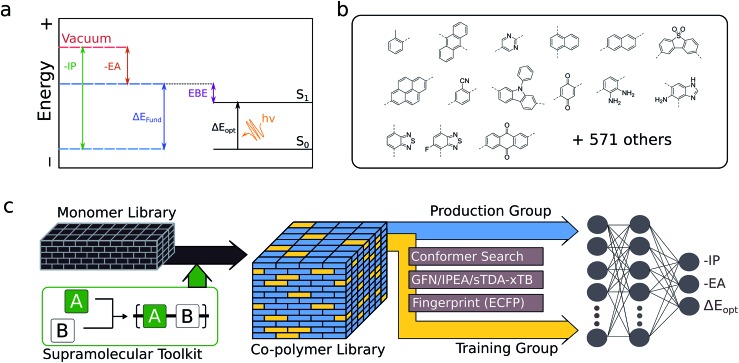
(a) Illustration of the relationships between the negative of the ionisation potential (–IP) and electron affinity (–EA), fundamental gap (Δ*E*_fund_), exciton binding energy (EBE) and optical gap (Δ*E*_opt_). (b) Examples of monomers used to construct the monomer library (15 shown out of 586). (c) Outline of the workflow used to generate optoelectronic training data for a random selection of ∼50 000 copolymer compositions from the total number of possible compositions. The resulting neural network model is used to predict the properties of the remaining ∼310 000 compositions.

Here we show how high-quality training data obtained *via* xTB, in combination with 2D molecular descriptors (in this case, Extended-Connectivity (Morgan) Fingerprints^40^), can be used to train a neural network model capable of the simultaneous, near-instant prediction of the key optoelectronic properties of copolymers with very high accuracy (RMSE < 0.12 eV). Using this model, we explore the binary copolymer property space spanned by a pool of 586 monomeric units that are compatible with Yamamoto, Suzuki–Miyaura or Stille coupling (see Fig. 1b for examples), generating around 350 000 possible unique copolymer structures. This library was compiled from commercially available aromatic dibromides and distannanes, as well as non-commercially available building blocks from the organic photovoltaics literature. With this large volume of data, we are able to identify general features of the property space of binary copolymers and their homopolymer counterparts, test the ideas behind common synthetic strategies used to yield low-optical-gap materials, and explore the extent to which polymer properties can be tuned through copolymerisation.

## Methodology

### Properties of interest and polymer models

The optoelectronic properties of a conjugated polymer may be characterised by the key quantities^41^ outlined in Fig. 1a. These are the ionisation potential (IP), the energy required to remove an electron from the polymer; the electron affinity (EA), the energy released upon adding an electron to the polymer; and the optical gap, the minimum energy at which the polymer absorbs light to form an interacting electron–hole pair (exciton). Two additional quantities may be derived from these: the fundamental gap, the energy required to form a completely non-interacting electron–hole pair; and the exciton binding energy, a measure of the interaction energy between the excited electron and hole in the exciton (the difference between the optical and fundamental gaps). Note that, throughout the text, we generally focus on the negative of IP and EA, (–IP and –EA), which map directly onto the commonly used HOMO (–IP) and LUMO (–EA) concepts which are often used as approximations to these quantities. Additionally, we approximate the optical gap as the lowest energy excitation (S_0_ → S_1_) for all polymers.

In line with previous work,^18^,^42^–^45^ we model polymer materials as long-chain oligomers, with the environment of an oligomer in the bulk polymer approximated in the xTB calculations by a dielectric continuum. In previous work we showed that such a model yields accurate –IP, –EA and optical gap values compared with experimental measurements derived from photoelectron spectroscopy^44^ and UV-vis absorption spectra.^18^,^45^

### Training data generation

The generation of training data follows a tiered strategy, where a relatively small, diverse subset of copolymers is used to calibrate the accurate trends in properties given by a family of semi-empirical methods to the absolute values given by DFT. Within this family of semi-empirical, density functional tight-binding methods, GFN-xTB^37^ is used for structural optimisation of the neutral polymers. For –IP/–EA calculations, we use an extension of the parent GFN-xTB method, IPEA-xTB,^38^ a variant of GFN-xTB especially parameterised by Grimme and co-workers for the calculation of –IP and –EA values. For optical gaps, we employ the tight binding simplified Tamm–Dancoff approximation (sTDA)^36^ applied to orbitals and orbital eigenvalues obtained from xTB (sTDA-xTB),^46^ an approach capable of ultrafast computation of entire UV-vis absorption spectra. All GFN-xTB and IPEA-xTB calculations were performed using the *xtb* code,^47^ while the sTDA results were obtained using the *stda* code.^48^ All GFN-xTB and IPEA-xTB calculations, but not sTDA calculations, used the generalised Born surface area solvation model, with the default parameters for benzene distributed with the *xtb* code, so as to approximate the environment of a polymer chain in an amorphous polymeric solid. The xTB –IP, –EA and optical gap values are calibrated to those predicted by B3LYP^49^–^52^ using a linear model and our previously published parameters for the low dielectric permittivity case.^35^

Structures for the xTB calculations are generated in a 3-step approach. Starting from a 2D simplified molecular-input line-entry system (SMILES)^53^ representation of each monomeric unit, linear polymer structures were generated using the Supramolecular Toolkit (*stk*),^54^,^55^ a Python library for the assembly, structure generation and property calculation of supramolecules, which takes base functionality from RDKit. *stk* allows for flexible copolymer formation from arbitrary monomer units, control over monomer sequence within repeat units, and the automatic generation of different structural isomers where asymmetric monomer units (*e.g.* 2,5 linked pyridine) are concerned. In all cases, we restrict repeat units to two monomer units and the polymer chains to 8 monomer units in total, a length that we have previously shown to provide approximately converged optoelectronic properties.^44^ Where asymmetric monomer units are concerned, we generate both possible ordered isomers. In a second step, a conformer search is performed using the stochastic Experimental-Torsion Distance Geometry with additional basic knowledge (ETKDG)^56^ method, where we typically generate 500 conformers per polymer. The resulting conformers undergo a subsequent optimisation and energy ranking procedure using the Merck Molecular Force Field (MMFF)^57^ as implemented in RDKit,^58^ where the lowest energy conformer according to MMFF is selected for the xTB calculations.

### Neural network training and evaluation

Although all xTB calculations are performed on long-chain oligomer models, we use trimers to generate molecular descriptors in the form of fixed-dimensional bit vectors using Extended-Connectivity Fingerprints (ECPF). These bit vectors are obtained directly from the 2D SMILES representations of each trimer using RDKit. Using trimers instead of the entire oligomer chain to obtain molecular fingerprints dramatically reduces the computational effort required for fingerprinting, while preserving all of the sub-structural information of the polymer. The use of 2D SMILES rather than representations of the 3D structures of the polymers is supported by the weak dependence of the optoelectronic properties of the polymer on the conformational degrees of freedom,^35^,^39^ already alluded to in the introduction (see also Fig. S1†). Though we explored different bit lengths and fingerprint radii, it may be assumed that results were obtained using a 2048 bit and radius 2 fingerprint, unless otherwise stated. The neural network itself has two hidden layers of 128 neurons each, using rectified linear (ReLu)^59^ activation functions throughout. To avoid overfitting, the neural network is regularised using dropout.^60^ Each of the training hyper-parameters, the dropout fraction, as well as the neural network architecture, were obtained by 100 iterations of a random search across the hyper-parameter space (for details, see the ESI†). The network was trained to minimise the mean absolute error (MAE) of the predicted IP, EA and optical gap values using the Adam optimisation algorithm as implemented in Tensorflow.^61^ The model was evaluated using a simple 50% train-test split of ∼50 000 polymer structures for which the target properties are calculated. The fingerprinting, model construction, and model training can be reproduced using a freely-available, easy-to-use Python interface.^62^

## Results and discussion

### Model generation and performance

The final model was obtained *via* a ‘data enrichment’ process, whereby predictions made for all polymers by the initial model were projected onto 2D property spaces (*e.g*. –IP *vs.* –EA). Areas towards the edge of these property projections with a low density of points (*i.e.* shallow –IP, deep –EA and low optical gap) were identified. Monomer units, which were statistically over-represented in these regions, were combined exhaustively with each other and the properties of the resulting copolymers calculated. A fraction (50%, approximately 900 additional examples) of the resulting data is then applied in re-training the neural network model. Here, this procedure is only conducted once, but it is conceivable that it could be performed over many iterations to generate more robust models from more limited training data. Fig. S2† shows the effect of this data enrichment process. Generally, we see that points at the extrema of the property projection plots tend to be exaggerated (*e.g.* –EA values are under-estimated) prior to re-training.

**Fig. 2:**
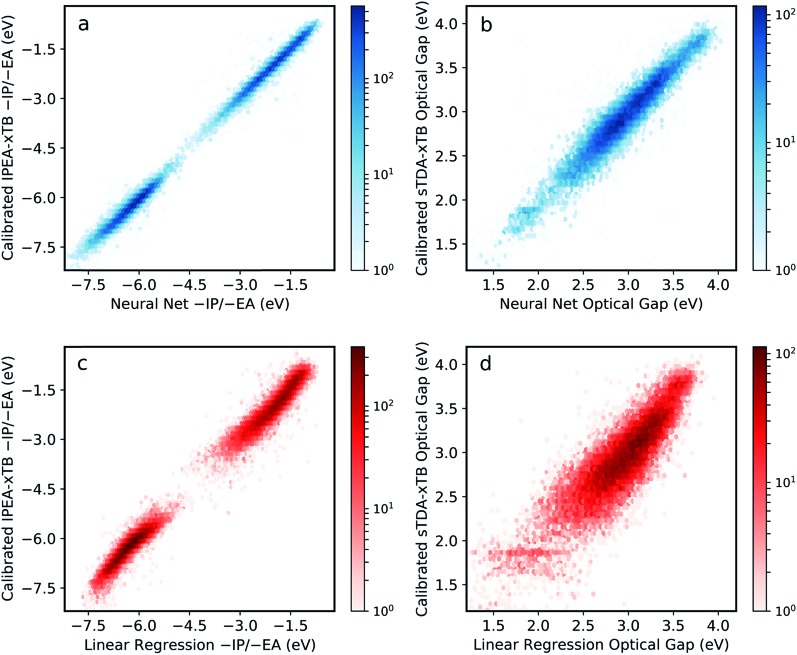
Performance of neural network model when predicting (a) –IP and –EA, (b) optical gap (S_0_ → S_1_ excitation energy) values derived from calibrated IPEA-xTB and sTDA-xTB, respectively shown as 2D histograms (dark red (high) – light red (low) density). For comparison, the performance of a linear regression model is also given (c and d). All properties correspond to copolymer compositions not used during the training phase.

The resulting neural network model clearly performs very well across the entire range of properties and property values, with root mean square error (RMSE) of less than 0.12 eV when predicting –IP, –EA and optical gap simultaneously (Fig. 2a and b). This represents a significant improvement in performance over previous attempts for polymers,^34^ and a far larger compositional space by several orders of magnitude. Comparing to a linear regression model obtained with an identical ECFP bit length and radius (Fig. 2c and d), we see that the neural network outperforms the linear model significantly for all properties (the linear regression model yields an RMS error of 0.30 eV overall). This comparison demonstrates that the neural network model captures some degree of non-linearity when mapping molecular substructures to optoelectronic properties. For high-throughput screening purposes, the neural network model accuracy is perhaps even greater than required, with absolute values as well as relative ordering of polymer properties adequately recovered. Further, high-throughput workflows, which rely on a cost-efficient method to screen very large number of structures, generally involve a post-large-scale-screening stage, where a promising subset of systems are taken forward and treated at a more computationally intensive level of theory. In this case, however, it appears that this step could effectively be negated by the inherent model accuracy.

Fig. S3† shows model performance when predicting differences in optical gap between isomers using different fingerprint bit lengths and radii. While we observe improvements in this quantity at longer bit lengths and radii, no significant improvement of the overall model performance is observed and, indeed, increasing these parameters may be detrimental to model generality. On the other hand, effects of monomer isomerism (in the case of asymmetric monomer units) are far better (albeit still roughly) captured at longer radii. This is consistent with the idea that distinctions between repeat unit isomers can only be made effectively when considering larger molecular fragments. In the future, some form of feature engineering could potentially be used to account for monomer isomerism more explicitly.

### Comparing the property space of homo and binary copolymers

The large and varied data set at our disposal means that we can empirically probe the optoelectronic property space of binary copolymers and how it differs from that of homopolymers. The optoelectronic property space is a 3D space spanned by vectors corresponding to a polymer's –IP, –EA and optical gap values. The fundamental gap is by definition equal to the difference between –IP and –EA and hence not a free parameter. Fig. S4† shows an image of this property space, showing that all polymers lie in an almost 2D plane embedded in the 3D space. The quasi-two-dimensional nature of the optoelectronic property space finds its origin in the fact that (i) in the limit of zero exciton binding energy, the optical gap would equal the fundamental gap and (ii) the predicted exciton binding energies (∼0.5–2 eV), while large compared to classical inorganic semiconductors, are small relative to the fundamental gap (∼2–6 eV, see Fig. S5†).

**Fig. 3:**
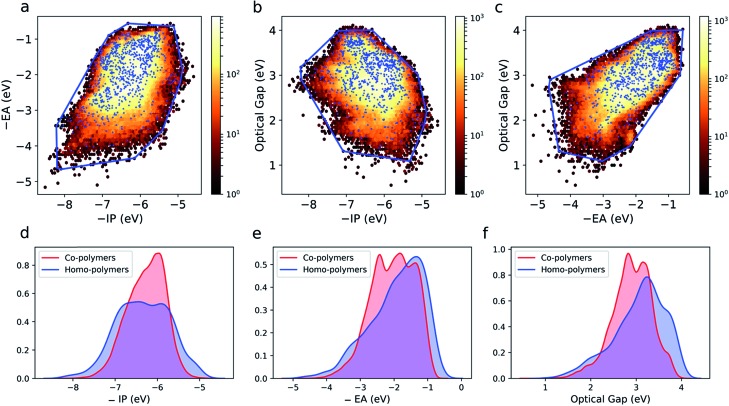
2D histograms of copolymer property spaces spanned by (a) –IP and –EA, (b) –IP and optical gap, (c) –EA and optical gap. In each case, the property space spanned by copolymers (dark red (low) – yellow (high) density) and homopolymers (blue dots) is shown. The property space enclosed by the homopolymers is also shown as a convex hull (blue line). Kernel density estimates (KDE) of (d) –IP, (e) –EA and (f) optical gap for both homo- and copolymers.

Fig. 3a–c shows projections of the 3D optoelectronic property space on 2D surfaces spanned by (i) –IP and –EA, (ii) –IP and optical gap, and (iii) –EA and optical gap, respectively, where we have drawn convex hulls enclosing all homopolymers in each case. Comparing these homopolymer convex hulls with the plotted points for the copolymers it appears that only a very small number – likely to be statistically insignificant for a dataset of this size – of copolymers lie outside of the property space spanned by homopolymers. The homopolymers also appear to sample the property space proportionally to the density of copolymers within a given subspace. This suggests that copolymerisation, at least in the case of ordered binary copolymers, does not allow access to additional regions of the optoelectronic property space not already sampled by the homopolymers. The density of points in the case of the copolymers is much larger though, conceptually allowing for more fine-grained property control. Further, we would like to emphasise that these observations may not hold for other properties (*e.g.* charge-transport properties) and more complex co-polymer repeat units (*e.g.* ternary and quaternary co-polymers). Finally, we note that, even if the vast majority of copolymers lie inside the homopolymer convex hulls, this does not necessarily mean that the properties of a specific copolymer lie in between those of the two corresponding homopolymers, as we will discuss later.

Fig. 3d–f shows kernel density estimates of the distributions of –IP, –EA and optical gap values for both the homo and copolymers. Here we see that the co-polymer property space spans a broad range of values, with significant numbers of materials present over a range of more than 4 eV for each property. It is clear that in all cases the copolymer distributions are more symmetrical than those of their homopolymer counterparts.

### Correlations between copolymer properties

The 2D projections in Fig. 3a–c shows that there are weak correlations between the different properties. In the case of –IP and –EA, binary copolymers and homopolymers with deep –IP values are likely to also have deep –EA values and *vice versa*. In the case of the optical gap, binary copolymers and homopolymers with small(er) optical gaps are more likely to have shallower –IP values. Similarly, the same polymers are more likely to have deeper –EA values. It is unclear if these correlations are evidence of some deeper relationship or merely result from the fact that the fundamental gap values of the polymers span a range of around 4 eV. Regardless, as we study a large range of monomers, and therefore copolymers, it is apparent that certain property combinations might be difficult to achieve (*e.g.* copolymers that both have a shallow –EA value and a small optical gap; copolymers with a shallow –IP value and a large optical gap) due to the absence of copolymers in these regions of property space. As these regions are also not sampled by the homopolymers, this is simply the result of practically all binary copolymers lying within the homopolymer convex hull.

### Emergence of copolymer properties and the donor–acceptor model

As briefly mentioned in the introduction, models that explain the copolymer optoelectronic properties in terms of the donor and acceptor properties of the monomeric building blocks have been proposed in the literature. In the same vein, we compare the optoelectronic properties of copolymers to their homopolymer counterparts formed from the same building blocks. The reason for comparing with homopolymers rather than monomers is two-fold. Firstly, we do not have direct access to the optoelectronic properties of the isolated building blocks *via* the neural network. Secondly, the direct comparison of optoelectronic monomer and copolymer properties is inherently fraught by the conflation of effects due to the electronic coupling between the different monomers and their polymerisation.

**Fig. 4:**
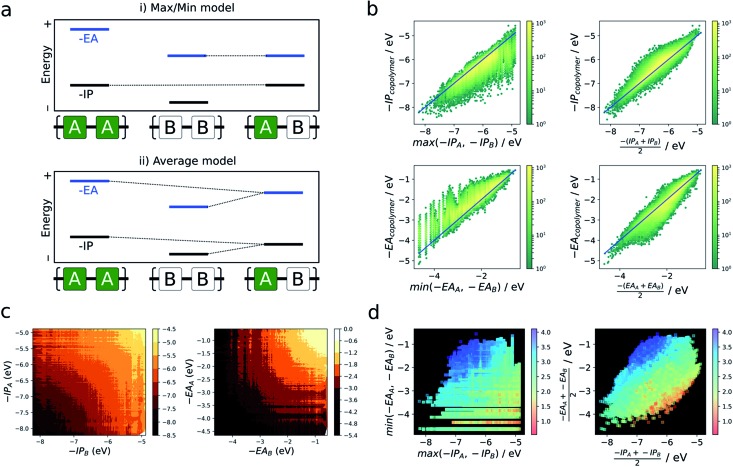
(a) Illustration of two simple models used to predict copolymer properties from those of its ‘parent’ homopolymers formed of its constituent monomers. (i) Max/min model (top), where the copolymer is assumed to inherit its –IP (–EA) from the parent homopolymer for which it is most shallow (deep). (ii) Average model, where the copolymer properties are averages of those of the parent homopolymers (bottom). (b) Results of applying each of these models to predict –IP and –EA of the copolymer database as 2D histograms (yellow (high) – green (low) density), where reference values are given by the neural network. (c) Contour plots of copolymer –IP and –EA as a function of parent homopolymer –IP/–EA. (d) Scatter plots of –IP & –EA values predicted for each model, coloured according to the optical gap values predicted by the neural network.

In the absence of a clear first principles model for this relationship, we employ two simple empirical models which explore two different regimes (i) a “max/min” model in which the –IP and –EA of the copolymer are predicted by the least negative (shallowest) –IP value and the most negative (deepest) –EA value of the relevant homopolymer pair, and (ii) an “averaging” model in which the –IP and –EA values are approximated by the arithmetic mean of the –IP and –EA values of the homopolymer pair (Fig. 4a).

Fig. 4b shows the performance of these models in terms of the –IP and –EA value of the copolymers. We observe that the averaging model performs well in terms of predicting the –IP and –EA values of the copolymers, with an RMSE of 0.16 eV overall. The max/min model performs less well (RMSE = 0.38 eV), while appearing to estimate a lower (upper) boundary to the –EA (–IP) value of a copolymer, reflecting the convex hull analysis in Fig. 2a–c. Additionally, we observe that the average model shows the largest deviation for copolymers where the difference between the –IP (or –EA) values of the homopolymer pair is large (see Fig. S6†), with a general over- and under-estimation of –EA and –IP, respectively. This is also consistent with the qualitatively curved contour lines shown in Fig. 4c, where, when the difference between –IP/–EA homopolymer values is large, the more positive –IP/more negative –EA homopolymer skews the resulting copolymer property further from a perfect average value. Conversely, where the difference between homopolymer values is small, the resulting copolymer properties are closer to the simple average value. Finally, as can be seen in Fig. S8† use of the averaging model can also qualitatively reproduce the convex hull picture shown in Fig. 2a. Overall, expressing copolymer properties as a simple average of ‘parent’ homopolymers appears to be an effective model for most polymers.

**Fig. 5:**
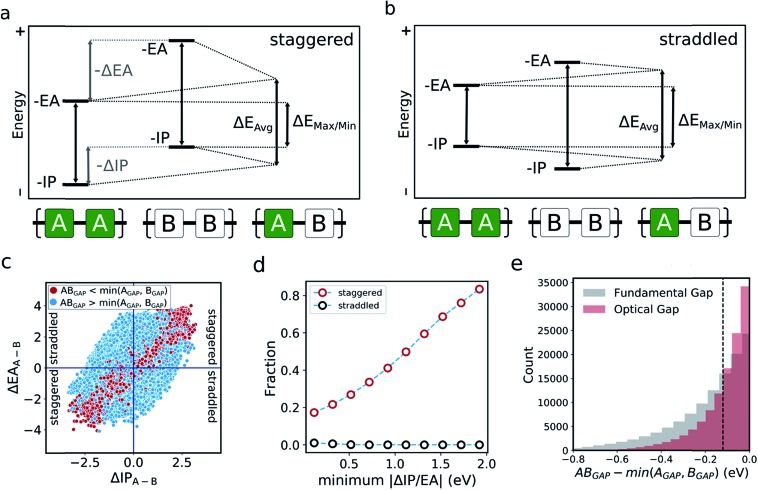
Two situations that arise where monomers have significantly different electronic properties. (a) ‘Staggered’ energy levels, where both the –IP and –EA values of one homopolymer are greater (or lesser) than those of the other. (b) ‘Straddled’ energy levels, where either the –IP or –EA values of one homopolymer are greater than those of the other. (c) Plot of whether a copolymer optical gap is less than (red) or greater than (blue) that of both related homopolymers, as a function of the difference between –IP and –EA homopolymer values. Quadrants related to ‘staggered’ and ‘straddled’ energy levels are highlighted. (d) Fraction of co-polymers within the staggered (red) and straddled (black) arrangements for which the observed optical gap is at least 0.12 eV lower than that of both related homopolymers as a function of the smallest of the differences between the IP and EA values of the related homopolymers. (e) Cumulative histogram of copolymers for which the optical gap/fundamental gap is less than that of both related homopolymers. Dashed line indicates overall RMSE of neural network model.

In the literature, the case for copolymerisation is often based on the ‘donor–acceptor’ strategy,^19^,^21^ where combining monomers with ‘donor’ and ‘acceptor’ qualities allows one to obtain copolymers with small(er) optical gaps. Here, we can use the large volume of data at our disposal to explore this concept and how it relates to the two empirical models discussed above. Indeed, the predictions made by the neural network identify some co-polymers for which the optical gap is lower than that of the two corresponding homopolymers (Fig. 5c). Specifically, we observe that ∼17 000 out of ∼350 000 copolymers studied have an optical gap that is at least 0.12 eV (the overall RMSE of the neural network) lower than that of the homopolymers. As can be seen from Fig. 5c, such copolymers generally correspond to cases where the related homopolymers have significantly different –IP and/or –EA values, and almost exclusively for cases where the –IP and –EA values of the two homopolymers are staggered with respect to one another (Fig. 5a). Conversely, when the –IP and –EA values of one homopolymer straddle the other (Fig. 5b), no reduction in optical gap upon copolymerisation is predicted. Furthermore, the likelihood of reducing optical gap through copolymerisation appears to increase with the extent to which the –IP and –EA values are staggered (Fig. 5d), which we rationalise through the concomitant decreasing likelihood of this effect being countered by differences in the exciton binding energy between homo and copolymers. Overall, accounting for the overall RMSE of the neural network, we find that in our dataset ∼100 000 out of the ∼350 000 copolymers are staggered by at least 0.12 eV, ∼17 000 of which display an optical gap reduction of at least 0.12 eV. In contrast, the –IP and –EA values of the copolymers strictly lie in between those of the two corresponding homopolymers when accounting for the RMSE of the neural network model.

One can explain the above observations by noting that, while the averaging model predicts that the fundamental gap of copolymers always strictly lies in between that of both corresponding homopolymers, and while it is very successful for most copolymers considered, there are copolymers that deviate considerably from its predictions. Such copolymers, as discussed above, tend to correspond to cases where the difference between the –IP (and/or –EA) values of the homopolymer pair is large (see Fig. 4c and S5†). In these cases the fundamental gap tends towards that predicted by the max/min model. A combination of this with a staggered arrangement of the –IP and –EA values of the two homopolymers then gives rise to a fundamental gap that is smaller than either of the homopolymers (see Δ*E*_max/min_ in Fig. 5a). As can be seen from Fig. 1a, this explanation translates directly to the case of the optical gap, as long as the exciton binding energies in the co and homopolymers are not sufficiently different. As such, the requirement for a staggered arrangement maps on to the intuitive donor–acceptor picture used in the experimental literature, but stresses that these labels are only really meaningful when considering pairs of monomers and their properties relative to one another.

Overall, these observations and their explanation lend both context and understanding to the donor–acceptor strategy proposed in the literature. With knowledge of the optoelectronic properties of homopolymers alone, we can provide a simple heuristic to predict promising combinations of monomers, which are likely to result in low optical gap materials. Specifically, for optical gap reduction to likely occur, not only should the –IP and –EA values of the two corresponding homopolymers be significantly different, but they should also be staggered, along the lines of Fig. 5a. This is strongly illustrated by Fig. 5d, which shows that for staggered cases with large –IP and –EA differences optical gap reduction is highly likely, while for straddled cases the odds of optical gap reduction are effectively zero. The same observation would also suggest that a likely side effect of reducing the optical gap is that the –IP and –EA values of the resulting copolymers will lie closer to those predicted by the max/min model than its averaging counterpart. As a result, such copolymers will likely combine relatively shallow –IP and deep –EA values, reducing their potential applicability in domains such as photocatalysis, where the alignment of the polymer potentials relative to those of other materials or solution half-reactions is crucial.

### Monomer topography of the property space

**Fig. 6:**
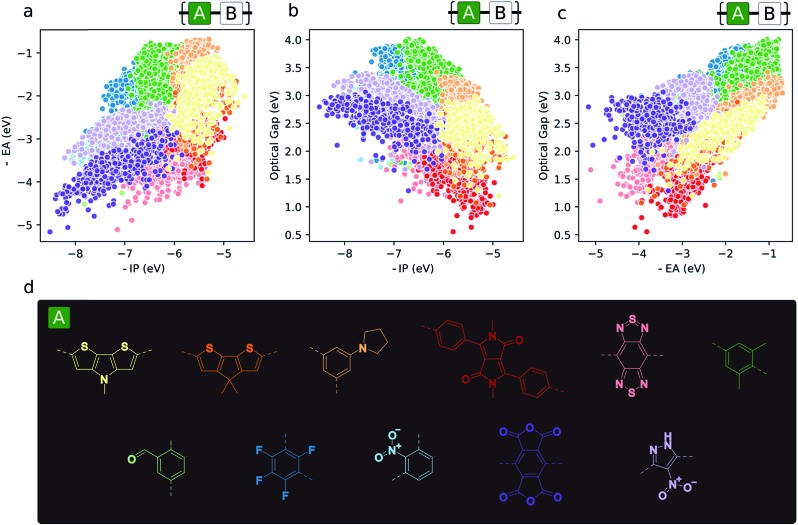
(a–c) 2D property spaces where the most prevalent monomer units within different regions are highlighted. (d) Colour key for monomer property sub-spaces shown in (a–c).

Aside from the general exploration of copolymer property space and the testing of models able to describe it, high-throughput calculations have the potential to guide synthetic efforts towards promising materials with properties amenable to certain applications. In the context of copolymers, this could mean either the identification of specific copolymer compositions or – perhaps more interestingly from synthetic accessibility and material morphology standpoints – monomers (*i.e.* dibromo compounds or diboronic acids/acid esters) – which target a particular region of property space. To illustrate this, we give examples of the most prevalent co-monomers in different regions of the property space (Fig. 6). From this analysis, we see the emergence of some common motifs found in, for example, the organic photovoltaics literature (namely, diketopyrrolopyrrole and benzothiadiazole), where smaller optical gaps are sought after to absorb more of the solar spectrum. Similarly, monomers that give rise to materials with deep –IP and not too deep –EA values, which are potentially attractive for water-splitting due to their large driving force for both proton reduction and water oxidation, contain electron-withdrawing substituents like –F and –NO_2_ (1,3-linked tetrafluorophenylene and 1,3-linked nitropyrazole). Additionally, these same monomers illustrate the idea that, due to the quasi-two-dimensional nature of the optoelectronic property space, choosing monomers that place –IP and –EA within a desired range also fixes the possible optical gap values to within the domain of possible exciton binding energy values. Finally, Fig. 6 also suggests that, for applications in which ohmic contacts between the polymer and an electrode are important, *e.g.* organic photovoltaics and organic light emitting diodes, to achieve barrierless charge injection or collection, the properties of the copolymer relative to an electrode can be anchored to a particular value range by copolymerisation with suitably chosen monomers.

## Conclusions

We have demonstrated that machine learning techniques – neural networks – can be used to resolve the optoelectronic property landscape of conjugated organic copolymers with very diverse monomer compositions. The neural network training is facilitated by the availability of large amounts of accurate, low-noise data derived from a tiered strategy based on calibrated density functional tight binding calculations, which display an accuracy on par with density functional theory. The property space generated by the neural network allows for the data-driven testing of simple models that link the properties of the constituent monomers of a copolymer to the properties of the copolymer itself. We observe that copolymerisation to make binary copolymers does not appear to allow access to regions of the optoelectronic property space not already sampled by the homopolymers, while allowing for more fine-grained property control. The large dataset at our disposal also facilitates the testing of common synthetic strategies such as using ‘donor’ and ‘acceptor’ monomers to construct low-optical-gap materials. Generally, despite the prevalence of this concept in the literature, we observe that this phenomenon is relatively rare. We predict that for a copolymer to have a significantly smaller optical gap than its related homopolymers, the potentials of these should be substantially offset and arranged in a staggered fashion. From here, one can imagine an application-specific, optimal balance between absolute value of the homopolymer potentials themselves and the extent to which they are staggered relative to one another that achieves ideal copolymer light absorption and redox properties. Additionally, we demonstrate that high-throughput methods could be used to identify promising monomers which target specific regions of property space.

## Conflicts of interest

There are no conflicts to declare.

## Supplementary Material

Supplementary information

Supplementary information

Supplementary information
