# Supplementary material for: Mapping binary copolymer property space with neural networks
Source: Chem Sci. 2019 Apr 1;10(19):4973–84. doi: 10.1039/c8sc05710a (PMC6530542; doi:10.1039/c8sc05710a)
Supplement: Supplementary file 1 [file SC-010-C8SC05710A-s001.pdf]

Electronic Supplementary Material (ESI) for Chemical Science.
This journal is © The Royal Society of Chemistry 2019

SUPPORTING INFORMATION

Mapping Binary Copolymer Property Space with Neural Networks

Liam Wilbraham[a], Reiner Sebastian Sprick[b], Kim E. Jelfs[c] and Martijn A. Zwijnenburg[a,*]

[a] *Department of Chemistry, University College London, 20 Gordon Street, London, WC1H 0AJ, UK.*

[b] *Department of Chemistry and Materials Innovation Factory, University of Liverpool, Crown Street, Liverpool, L69 7ZD, U.K.*

[c] *Department of Chemistry, Imperial College London, Molecular Sciences Research Hub, White City Campus, Wood lane, London, W12 0BZ, UK.*

Email of corresponding author: m.zwijnenburg@ucl.ac.uk

Table S1. Supporting files associated with this work.

training-data.csv	SMILES strings used to create fingerprints, associated properties calculated using (GFN/IPEA/sTDA)-xTB
neural-network-predictions.csv	IP, EA and optical gap values predicted by the neural network, trained for 20 epochs using the parameters shown in Table S1
neural-network-model.h5	Trained neural network model

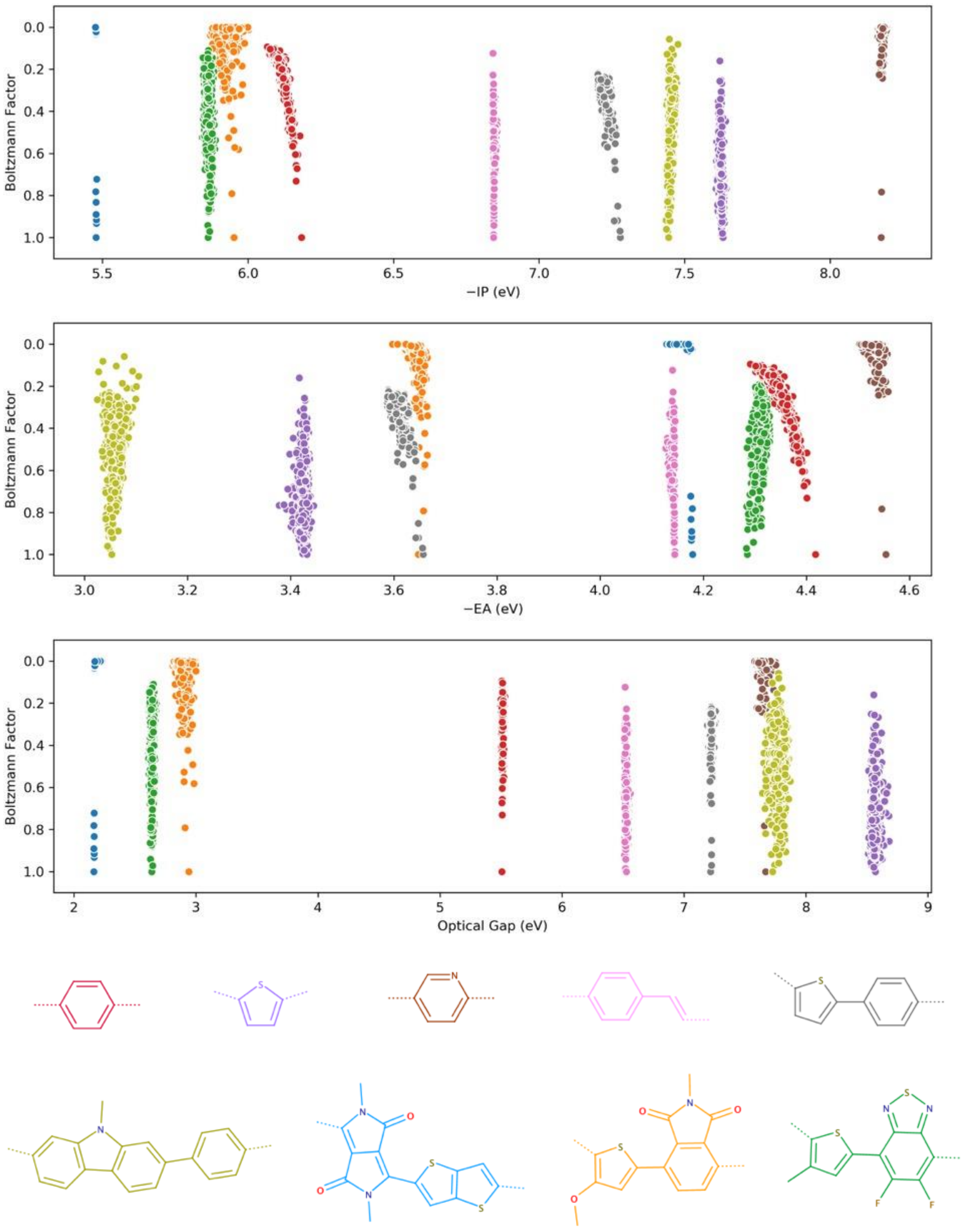

Figure S1. Sensitivity of properties to co-polymer conformation for a subset of structures. In each case, 500 conformers were sampled. The Boltzmann factor of each conformer relative to the lowest-obtained conformer is also given.

Table S2. Neural network model and training parameters obtained through random search. Random search was conducted using a freely-available python module that relies on Tensorflow and Keras. (https://github.com/ZwijnenburgGroup/pychemlp).

Number of hidden layers	2
Neurons per layer	128
Dropout fraction on input layer	40%
Dropout fraction on hidden layers	50%
Activation functions (all)	ReLu
Batch size	256
Optimizer	Adam
Loss function	Mean absolute error
Learning rate	0.001
Epochs	20

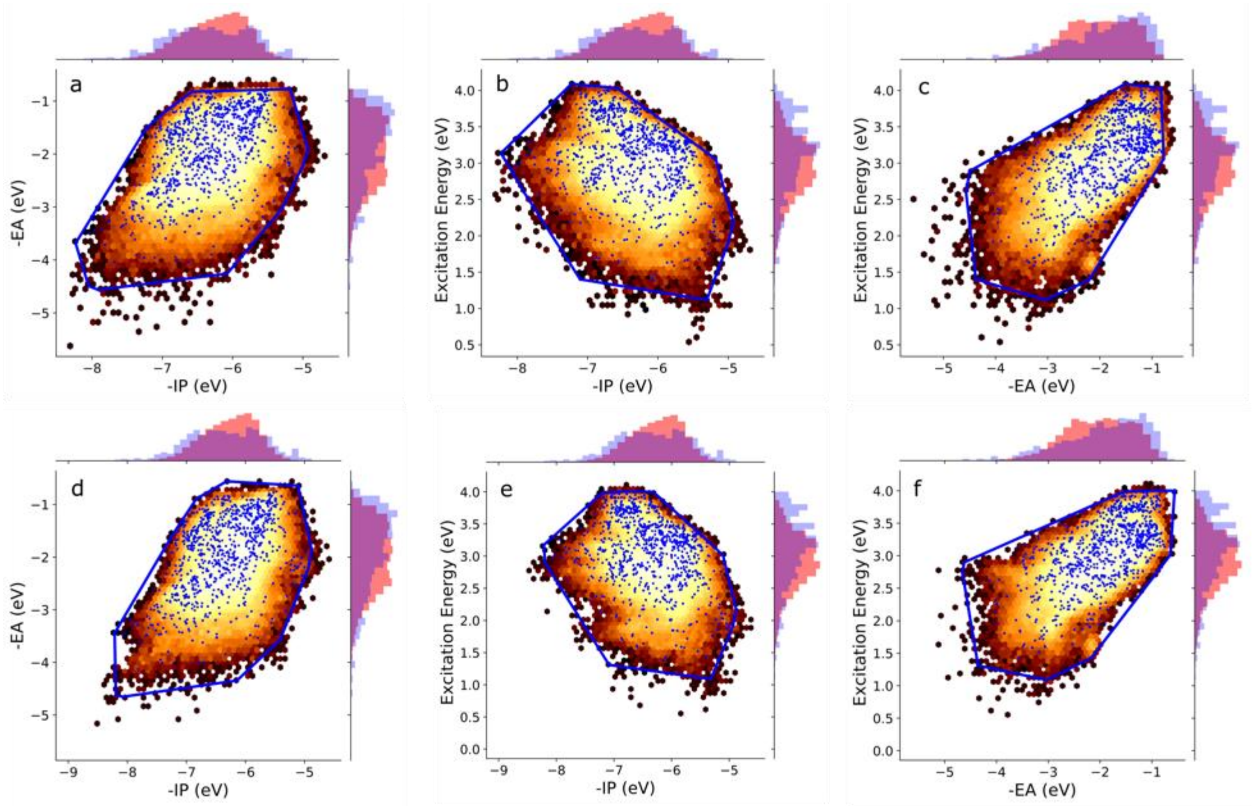

Figure S2. 2D histograms of copolymer property spaces spanned by –IP and –EA, –IP and excitation energy, –EA and excitation energy prior to (a-c) and after (d-f) data enrichment retraining process. In each case, the property space spanned by copolymers (dark red (low) – yellow (high) density) and homopolymers (blue dots) is shown.

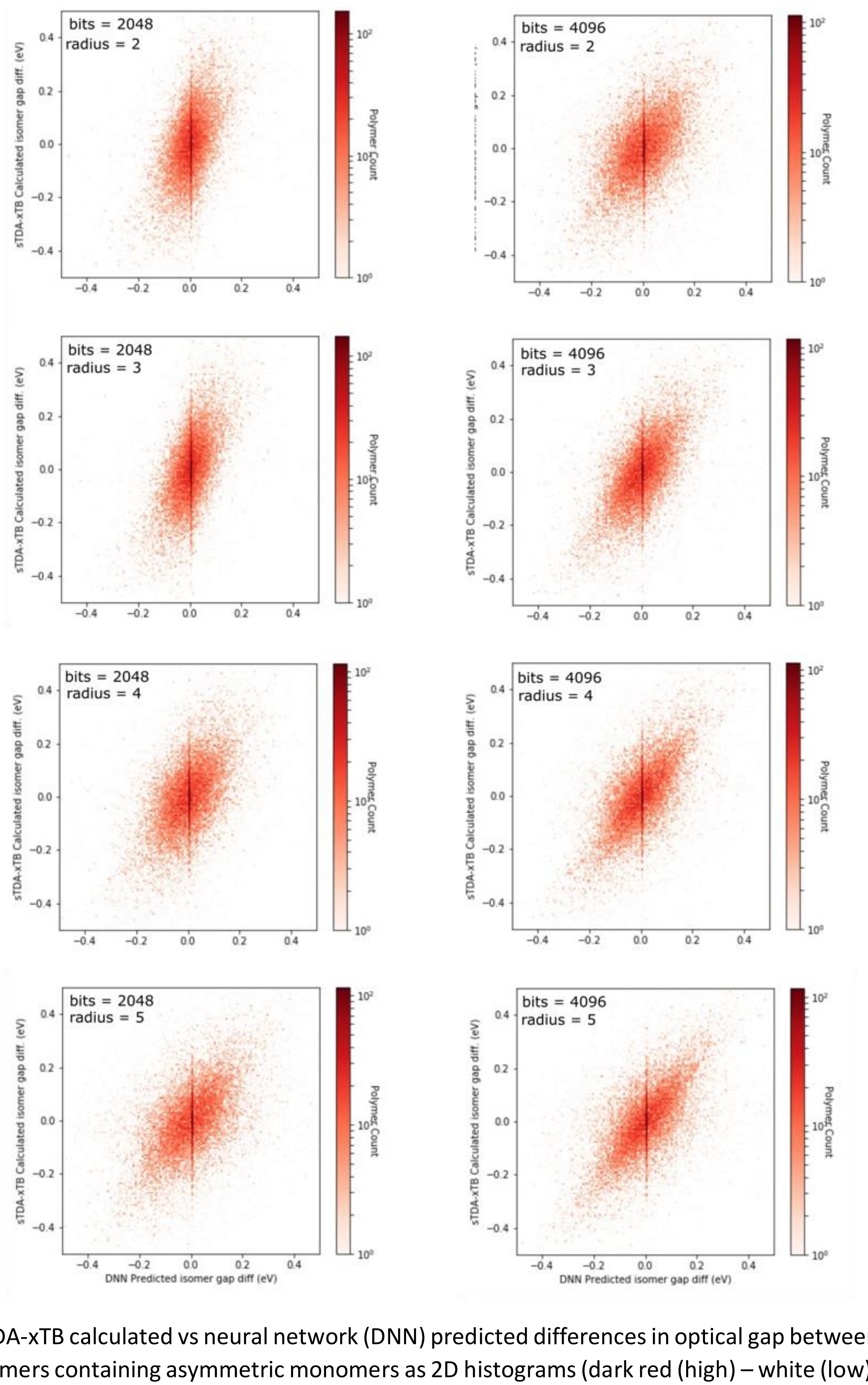

Fig S3. sTDA-xTB calculated vs neural network (DNN) predicted differences in optical gap between isomers of co-polymers containing asymmetric monomers as 2D histograms (dark red (high) – white (low) density). The radius and number of bits used to construct Morgan fingerprints is indicated.

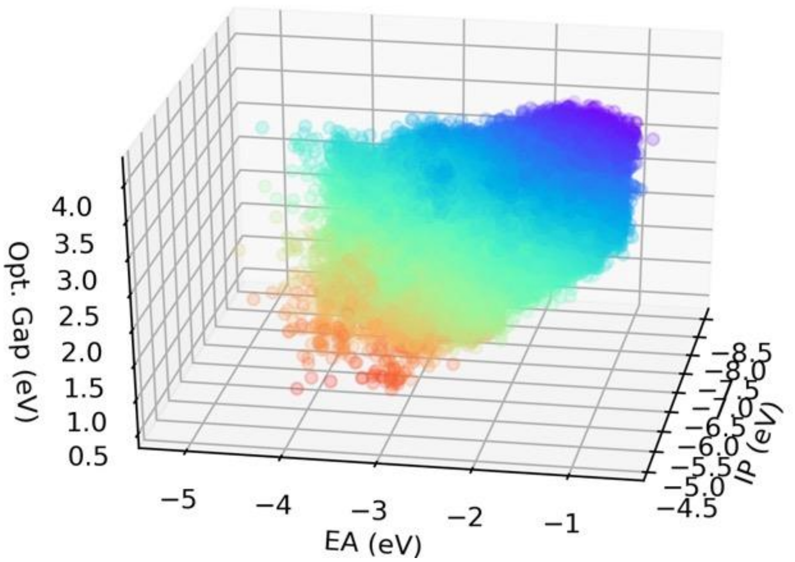

Figure S4. 3D representation of property space spanned by –IP, −EA and optical gap. Color scheme represents optical gap value (low, red – high, blue).

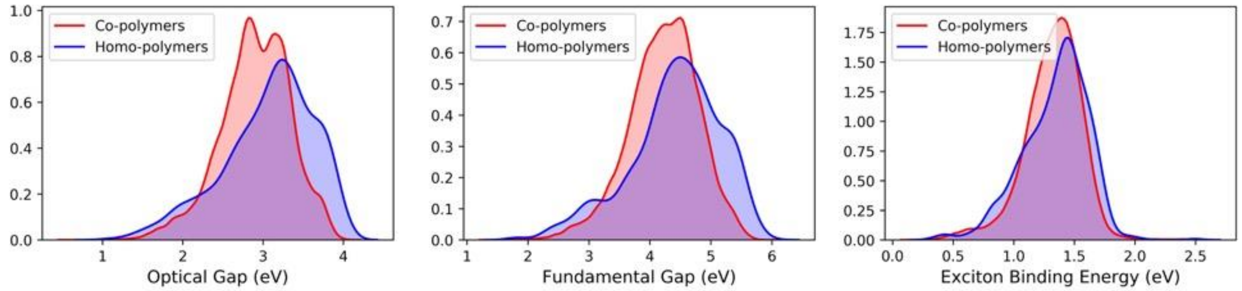

Figure S5. Kernel density estimates of optical gap, fundamental gap and exciton binding energy for both homo- and co-polymers as predicted by the neural network.

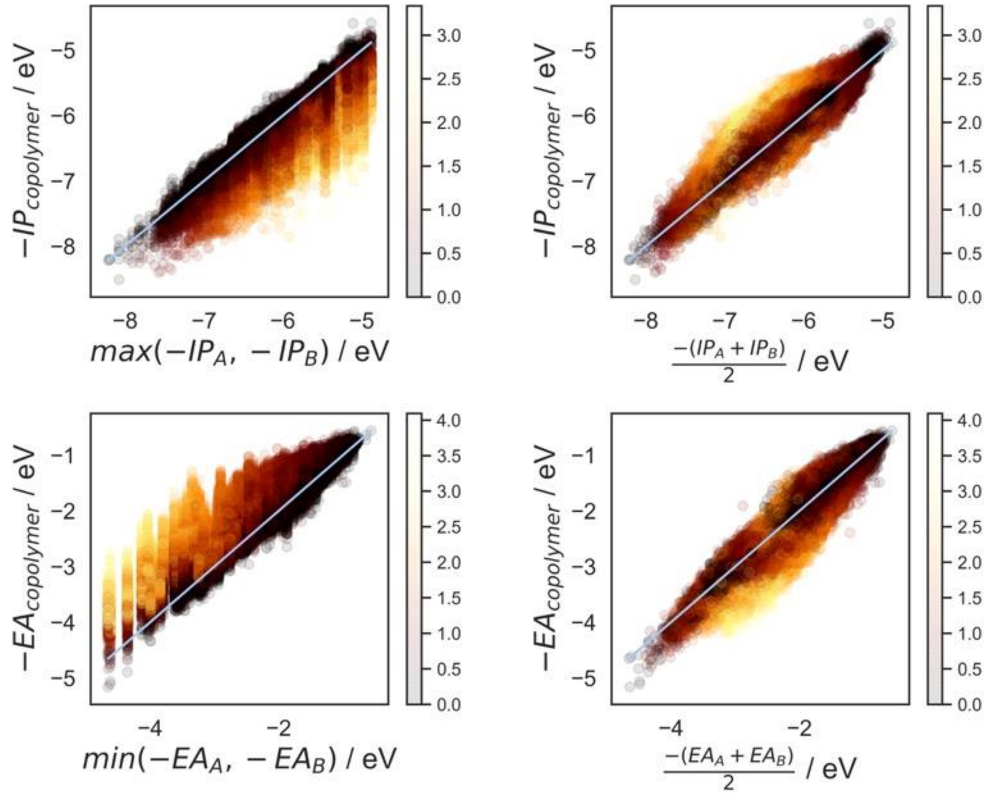

Figure S6. Max/Min and average models applied to predict –IP and –EA of the copolymer database, where reference values are given by the neural network. Colour scheme is the difference between –IP and –EA of each of the related homopolymers.

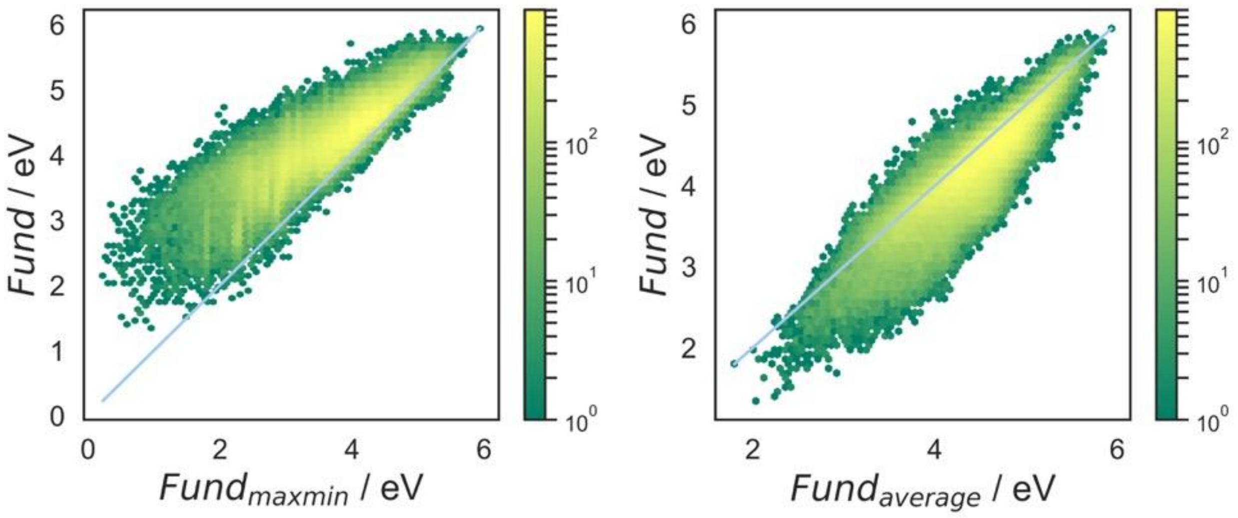

Figure S7. Max/Min and average models applied to predict fundamental gaps of the copolymer database as 2D histograms (yellow (high) – green (low) density), where reference values are given by the neural network.

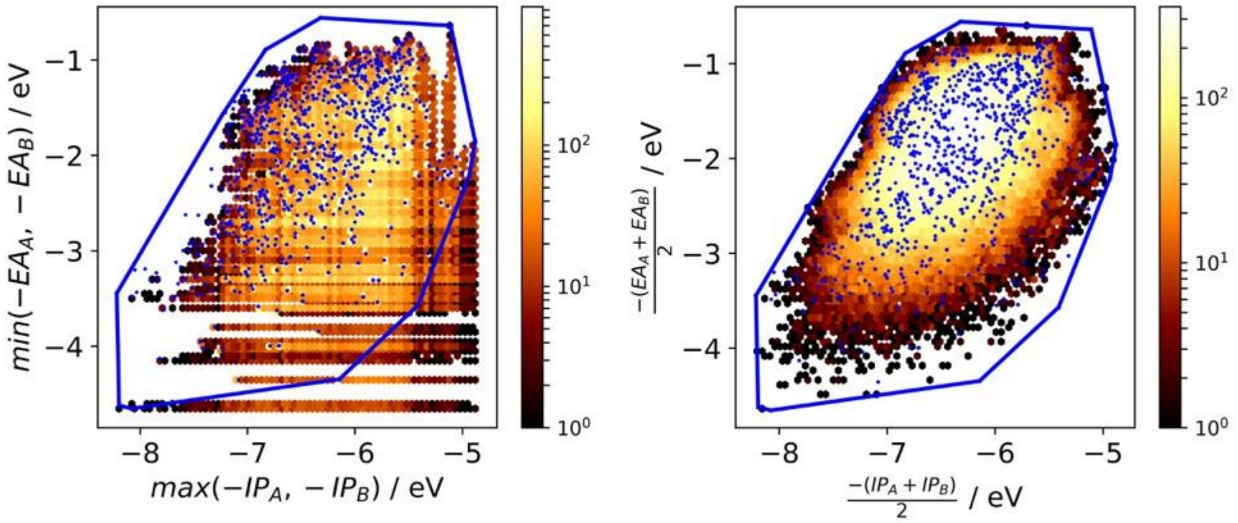

Figure S8. Property projections generated by using the max/min (left) and average (right) models to predict –IP and –EA of the copolymer database as 2D histograms (dark red (low) – yellow (high) density). Homopolymer values (blue) are those predicted by the neural network, the space spanned by their properties is enclosed by a convex hull, in analogy to Fig. 1 in the main text.

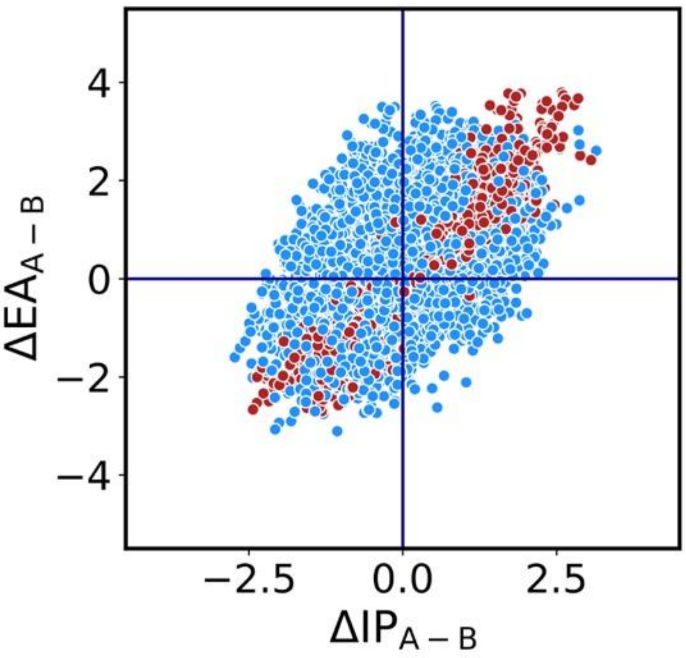

Figure S9. For copolymers for which the related homopolymers contain para-linked monomers (i.e. are fully-conjugated), plot of whether a copolymer optical gap is less than (red) or greater than (blue) that of both related homopolymers, as a function of the difference between –IP and –EA homopolymer values.
